# Exercise intensity regulates cytokine and klotho responses in men

**DOI:** 10.1038/s41387-020-00144-x

**Published:** 2021-01-07

**Authors:** Roeland J. W. Middelbeek, Piryanka Motiani, Nina Brandt, Pasquale Nigro, Jia Zheng, Kirsi A. Virtanen, Kari K. Kalliokoski, Jarna C. Hannukainen, Laurie J. Goodyear

**Affiliations:** 1 Section on Integrative Physiology and Metabolism, Joslin Diabetes Center, Boston, MA USA; 2 Beth Israel Deaconess Medical Center, Harvard Medical School, Boston, MA 02215 USA; 3 Turku PET Centre, University of Turku, 20521 Turku, Finland; 4 Turku PET Centre, Turku University Hospital, 20521 Turku, Finland; 5 Brigham and Women’s Hospital, Harvard Medical School, Boston, MA 02115 USA

**Keywords:** Homeostasis, Type 2 diabetes

## Abstract

**Background:**

Short-term exercise training programs that consist of moderate intensity endurance training or high intensity interval training have become popular choices for healthy lifestyle modifications, with as little as two weeks of training being shown to improve cardiorespiratory fitness and whole-body glucose metabolism. An emerging concept in exercise biology is that exercise stimulates the release of cytokines and other factors into the blood that contribute to the beneficial effects of exercise on metabolism, but whether these factors behave similarly in response to moderate and high intensity short term training is not known. Here, we determined the effects of two short-term exercise training programs on the concentrations of select secreted cytokines and Klotho, a protein involved in anti-aging.

**Methods:**

Healthy, sedentary men (*n* = 22) were randomized to moderate intensity training (MIT) or sprint intensity training (SIT) treatment groups. SIT consisted of 6 sessions over 2 weeks of 6 × 30 s all out cycle ergometer sprints with 4 min of recovery between sprints. MIT consisted of 6 sessions over 2 weeks of cycle ergometer exercise at 60% VO_2peak_, gradually increasing in duration from 40 to 60 min. Blood was taken before the intervention and 48 h after the last training session, and glucose uptake was measured using [^18^F]FDG‐PET/CT scanning. Cytokines were measured by multiplex and Klotho concentrations by ELISA.

**Results:**

Both training protocols similarly increased VO_2peak_ and decreased fat percentage and visceral fat (*P* < 0.05). MIT and SIT training programs both reduced the concentrations of IL-6, Hepatocyte Growth Factor (HGF) and Leptin. Interestingly, MIT, but not SIT increased monocyte chemoattractant protein-1 (MCP-1) concentrations, an exercise-induced cytokine, as well as Klotho concentrations.

**Conclusion:**

Short-term exercise training at markedly different intensities similarly improves cardiovascular fitness but results in intensity-specific changes in cytokine responses to exercise.

## Background

Short-term exercise training for as little as two weeks exerts multiple beneficial effects on the body, including improving whole-body glucose homeostasis and increasing insulin-stimulated glucose uptake in skeletal muscle and subcutaneous white adipose tissue^1–5^. The standard approach for short-term training programs has generally consisted of moderate-intensity training, defined as continuous bouts of exercise for 45–60 min at 70–75% of VO_2peak_. A more recently developed exercise training paradigm consists of high-intensity interval training (HIIT). HIIT programs are relatively short, vigorous bouts of exercise lasting for 1–4 min that elicit 80–100% of maximal heart rate, interspersed with episodes of rest or exercise with minimum intensity^6^. These HIIT sessions typically last 20–25 min and are generally performed 2–3 times per week. HIIT protocols performed with exceptionally high intensity (>100% of VO_2max_, 30 s/bout), are categorized as sprint interval training (SIT). While short-term moderate intensity training, high intensity interval training, and sprint interval training may induce systemic health benefits, the circulating factors contributing to the health benefits of these exercise training programs are not known.

Over the last decade an emerging concept in exercise biology is that in response to exercise, skeletal muscle and other organs initiate tissue-to-tissue crosstalk by the secretion and release of circulating factors^7,8^. These exercise-stimulated factors can include proteins, peptides, hormones, metabolites, and cytokines. Cytokines are ~5–20 kDa polypeptide proteins that play an important role in cell signaling and may mediate tissue-to-tissue cross-talk^9^. One of the most studied cytokines that has largely been investigated in the context of circulatory responses to a single bout of exercise is interleukin 6 (IL-6)^10^. The major source of IL-6 under resting conditions is macrophages and mature adipocytes from white adipose tissue^11^. IL-6 is also produced in white blood cells and skeletal muscle^12^. Studies in human subjects established that a single bout of moderate intensity exercise increases circulating IL-6 concentrations^13^, while an endurance exercise training program may reduce IL-6 concentrations^14^ or decrease the magnitude of the acute exercise IL-6 response^15^. The effects of short-term exercise training at different intensities on IL-6 concentrations have not been studied.

Another well-established cytokine involved in multiple biological functions is the small polypeptide of ~13 kDa monocyte chemoattractant protein 1 (MCP-1), also referred to as chemokine (C–C motif) ligand 2 (CCL2)^16^. MCP-1 plays a role in monocyte and T-cell recruitment^17,18^, inducing angiogenesis in endothelial cells^19^, and enhancing wound healing^20^. Incubation of primary human skeletal muscle cells with MCP-1 reduced insulin signaling and insulin-stimulated glucose uptake^21^. Conditioned media from human adipocytes contained MCP-1, suggesting adipose tissue can secrete MCP-1 that can exert effects in other organs^21^. MCP-1 has also been studied in the context of exercise. A single bout of HIIT exercise increased circulating MCP-1 concentrations in young men^22^ whereas in another study, MCP-1 concentrations did not change after two weeks of HIIT training^23^. Two weeks of moderate intensity training in obese subjects also did not change MCP-1 concentrations^24^. These data suggest that MCP-1 concentrations are responsive to an acute bout of exercise, but whether short-term exercise training at different intensities regulates MCP-1 concentrations has not been determined.

While exercise regulates the concentrations of circulating cytokines, exercise may also regulate the concentrations of secreted proteins with a higher molecular weight, an example of which is Klotho. Klotho is a transmembrane protein of ~130 kDa, and, as a product of alternative splicing, circulating soluble Klotho with a weight of ~70 kDa may exert multiple systemic effects on distant organs in an endocrine manner^25^. Secreted Klotho has putative enzymatic activity modulating glycoproteins, resulting in the removal of terminal sialic acids from N-linked glycans^25^. Secreted Klotho suppresses the activity of insulin and insulin-like growth factor-1 (IGF-1)^26^ by suppressing ligand-stimulated autophosphorylation of insulin and IGF-1 receptors^25^. Klotho is involved in aging, as a transgenic mouse model overexpressing Klotho showed an extended lifespan compared to wild-type mice^26^. Interestingly, soluble Klotho concentrations are increased with an acute bout of exercise in men and women^27,28^, and 12 weeks of moderate aerobic exercise training increases Klotho concentrations in post-menopausal women^29^. The effects of short-term exercise training, and the effects of exercise training at different intensities on soluble Klotho concentrations are not known.

To understand the systemic effects of short-term exercise training programs at two different intensities, we determined the effects of a moderate intensity training program and sprint interval training program for two weeks on the regulation of numerous cytokine and Klotho responses in men. Our data show that despite similar adaptations in cardiorespiratory fitness, only moderate intensity training, and not sprint interval training, increases the concentrations of MCP-1 and Klotho, demonstrating training intensity-specific regulation of circulating factors.

## Subjects and methods

### Study subjects

Twenty eight healthy middle‐aged sedentary men were recruited and randomized into either sprint interval training or moderate intensity continuous training. Of the initial 28 subjects, cytokine analysis was performed on 22 subjects (*n* = 12 for SIT, *n* = 10 for MIT), due to the availability of pre-training and post-training specimens^1,29^. Inclusion criteria consisted of: age 40–55 years; body mass index 18.5–30 kg/m^2^; and VO_2Peak_ up to 40 mL/kg/min. The study was approved by the local ethics committee of the Hospital District of Southwest Finland, Turku (decision 95/180/2010 §228) and was carried out in compliance with the Declaration of Helsinki. Informed consent was obtained prior to any study procedures. The Institutional Review Board of the Joslin Diabetes Center reviewed the biospecimen analysis plan. This study is part of a larger study, registered at clinicaltrials.gov (#NCT01344928), and part of the presented data have been published^1^. The recruitment process, inclusion and exclusion criteria, and study design have been previously described in detail^1,30^.

### Exercise training intervention, VO_2_peak test, and indirect calorimetry

Subjects performed either 6 sprint interval training sessions which consisted of 6 × 30 s all-out cycle ergometer sprints with 4 min of recovery between sprints or 6 moderate intensity continuous training (MIT) sessions which consisted of ergometer cycling at a constant intensity of 60% VO_2_peak and cycling cadence of 60 revolutions per minute, initially 40 min, increasing to 60 min by session 3–6 over a 2-week period^1^. All sessions were monitored and conducted in an exercise laboratory. VO_2peak_ was determined during a graded exercise capacity test 1 week before the exercise intervention and ~96 h after the last exercise session using the cycling ergometer test (Ergoline 800 s; VIASYS Healthcare, Germany) as described^1,30^. The two training programs were not designed to have similar workloads, and primarily due to the shorter total exercise time, SIT produced less workload than MIT^31^.

### Body composition and PET scanning

Using magnetic resonance imaging, body composition was determined, and abdominal subcutaneous and visceral adipose tissue masses were calculated^1^. Bioimpedance monitoring (InBody 720; Mega Electronics, Kuopio, Finland) was used to measure percentage body fat.

2-deoxy-2-[^18^F]fluoro-D-glucose ([^18^F]FDG) positron emission tomography/computed tomography (PET/CT) images were acquired by using the GE Discovery TM ST System (General Electric Medical Systems, Milwaukee, Wisconsin). Participants fasted ≥12 h and avoided physical activity, and caffeine and alcohol for ~48 h prior to the PET/CT scans^1^.

### Cytokine and klotho measurements

Blood was drawn before the intervention and 48 h after the last exercise session and processed for serum. Pre-training and post-training blood samples were available for analysis for a total of 22 subjects (12 sprint interval trained and 10 moderate intensity trained subjects). Serum cytokine concentrations of nerve growth factor (NGF), interleukin 6 (IL-6), interleukin 8 (IL‐8), Leptin, hepatocyte growth factor (HGF), monocyte chemoattractant protein 1 (MCP‐1), and tumor necrosis factor (TNF)‐α were analyzed using the Adipokine Magnetic Bead Panel 2 (Cat#HADK2MAG‐61 K; Millipore, Billerica, Massachusetts) on the Luminex‐Multiplex analyzer (Millipore). Klotho was measured using an ELISA kit (Human Klotho ELISA Kit, NeoBioLab, (#HK0034), according to the manufacturer’s instructions.

### Statistical methods

Descriptive statistics are provided as model‐based means and 95% confidence intervals (CIs). Logarithmic transformation was carried out for NGF, IL-6, insulin, and TNF-α. Statistical analyses were performed with hierarchical linear mixed models compound symmetry covariance structure, including 1 within‐factor (training; before and after intervention in the whole group) interaction term (training*intensity; the sprint interval training and moderate intensity training groups behaved differently for the change in variable, with significant differences between the training intensities). Missing data points were accounted for by restricted maximum likelihood estimation within the linear mixed models. Correlation analyses were performed between the variables on a whole‐group level (*n* = 22) and subgroups using Pearson’s correlation. All values are reported as model‐based mean (SAS least squares means) values from all of the variables measured before and after training. *P* values < 0.05 were taken to indicate statistical significance. The analyses were performed using SAS System, version 9.3 for Windows (SAS Institute Inc., Cary, North Carolina).

## Results

### Baseline characteristics of subjects and effects of short-term training

Subjects were all healthy, sedentary males, age 48 ± 5 years with a BMI of 26 ± 2.3 kg/m^2^, and VO_2peak_ of 34.2 ± 4.2 mL/kg/min (Table 1). Subjects were randomized to the sprint interval training (SIT) or moderate intensity training (MIT) intervention. There were no significant differences between subject groups at baseline, including age which was 48.2 years for both training interventions (Table 1). The short-term exercise training interventions for a total of six sessions over a two-week period similarly increased aerobic capacity, as determined by VO_2peak_ (Table 1). Whole-body fat percentage and visceral fat and waist/hip ratio were also similarly decreased after both the sprint interval training and moderate intensity training interventions, and fat free mass increased after the training intervention in both groups (Table 1). These data demonstrate that only two weeks of sprint interval training and moderate intensity training can induce improvements in cardiorespiratory fitness and body fat mass, and that there are similar responses to the two types of training.

**Table 1:** Descriptive statistics and results of two-way analysis of variance for characteristics of sprint interval training (SIT) and moderate intensity training (MIT) subjects.

	SIT (*n* = 12)		MIT (*n* = 10)		Training	Training*intensity
	Pre	Post	Pre	Post	*P* value	
VO_2Peak_ (ml/kg/min)	34.5 [31.9;37.1]	36.0 [33.4;38.6]	33.9 [31.0;36.7]	35.1 [32.3;38.0]	0.002	0.72
Body mass (kg)	84.1 [79.1;89.0]	83.5 [78.5;88.5]	84.8 [79.4;90.3]	84.7 [79.3;90.1]	0.19	0.40
BMI (kg/m^2^)	26.0 [24.5;27.4]	25.8 [24.3;27.2]	26.2 [24.7;27.8]	26.2 [24.6;27.8]	0.19	0.39
Whole body fat (%)	22.9 [20.6;25.3]	21.9 [19.6;24.2]	22.7 [20.2;25.3]	22.3 [19.7;24.8]	0.007	0.27
Subcutaneus fat (kg)^	4.1 [3.4;5.0]	4.0 [3.4;4.8]	4.2 [3.4;5.1]	4.1 [3.4;5.0]	0.08	0.25
Visceral fat (kg)^	2.9 [2.0;4.0]	2.7 [2.0;3.8]	2.4 [3.4;1.6]	2.3 [3.3;1.6]	0.02	0.97
Waist/Hip ratio	1.0 [0.9;1.0]	0.9 [0.9;1.0]	1.0 [0.9;1.0]	1.0 [0.9;1.0]	0.11	0.07
Fat free mass (kg)	64.7 [61.6;67.7]	65.1 [62.1;68.1]	65.3 [62.0;68.6]	65.6 [62.3;68.9]	0.047	0.59

The *p*-value for ‘Training’ shows the differences after training as a whole group, ‘Training*intensity’ demonstrates an interaction between the training intensities. All data are presented as model based means [95% confidence interval, CI]. Logarithmic transformation has been done to the variables with ^. The values are LS means translated into original unit. BMI (Body mass index), VO_2peak_ (aerobic capacity).

### Short-term exercise training reduces IL-6, HGF, and Leptin concentrations, and moderate intensity exercise increases MCP-1 and Klotho concentrations

**Fig. 1:**
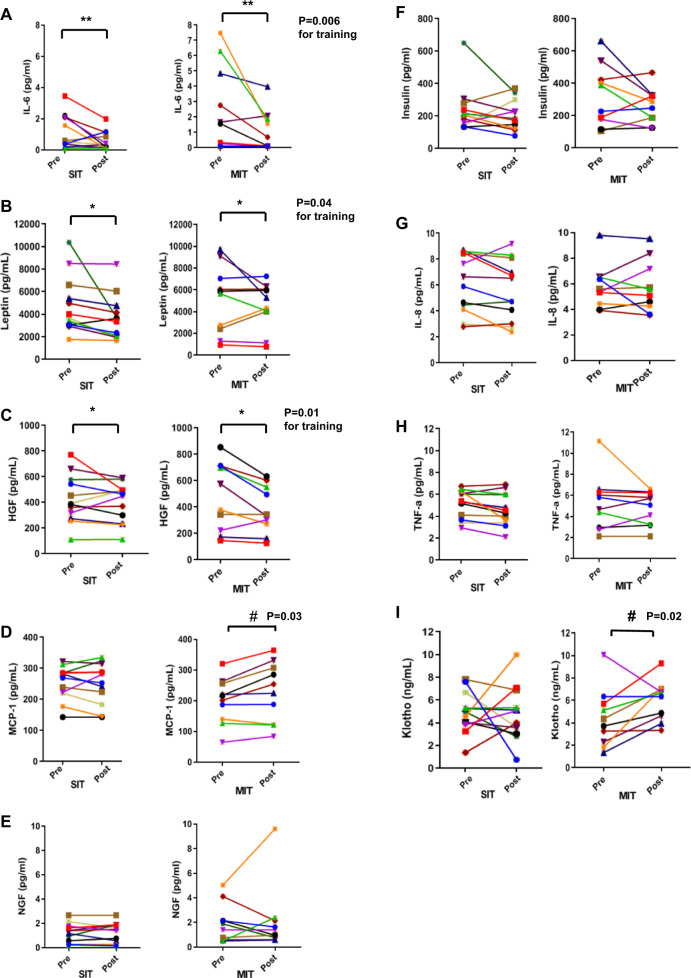
Effect of sprint interval training and moderate intensity training on cytokine concentrations. Changes in IL-6 (**a**), Leptin (**b**), HGF (**c**), MCP-1 (**d**), NGF (**e**), Insulin (**f**), IL-8 (**g**), TNF-α (**h**), and Klotho (**i**) pre- and post-training. All concentrations are in pg/ml, except for Klotho, which is ng/ml. *P < 0.05 for differences pre-training and post-training. **P < 0.01 for differences pre-training and post-training. ^#^*P* < 0.05 for differences between exercise training intensities. Data for NGF, IL-6, Insulin and TNF-α were log transformed. *N* = 9–12 per group.

The beneficial effects of exercise training may be mediated by a number of small molecular weight cytokines mediating tissue-to-tissue crosstalk. IL-6 is a well-studied myokine that has both pro-inflammatory and anti-inflammatory properties. Except for one subject in each group, all subjects decreased IL-6 concentrations after short-term training (Table 2, Fig. 1a). Exercise training reduced IL-6 concentrations in healthy men by 49% for sprint interval training and by 11% in moderate intensity training, although this reduction was not statistically significant between exercise training intensities. These data demonstrate that in contrast to the effect of acute exercise to increase IL-6, both moderate and high intensity short-term exercise training reduce IL-6 concentrations. These data also show that two weeks of short-term training induces similar reductions in IL-6 concentrations as seen after long-term training, independent of training intensity^14^.

**Table 2:** Descriptive statistics and results of two-way analysis of variance for cytokine concentrations of sprint interval training (SIT) and moderate intensity training (MIT) subjects.

	SIT (*n* = 12)		MIT (*n* = 10)		Baseline	Training	Training*intensity
	Pre	Post	Pre	Post	*P* value		
Log IL-6	0.63 [0.28.1.43]	0.32 [0.16;0.80]	1.04 [0.43;2.53]	0.44 [0.18;1.06]	0.44	0.006	0.54
Leptin	4814.6 [3349.9;6279.3]	3673.5 [2208.8;5138.2]	5079.9 [3475.4;6684.4]	4516.2 [2911.7;6120.7]	0.83	0.04	0.48
HGF	425.8 [308.9;542.6]	400.8 [283.9;517.6]	481.0 [353.0;609.0]	381.3 [253.3;509.3]	0.57	0.01	0.12
MCP-1	253.6 [209.7;297.5]	251.6 [207.7;295.5]	199.5 [151.4;247.6]	228.1 [180.0;276.2]	0.06	0.06	0.03
Log NGF	0.91 [0.52;1.58]	0.86 [0.50;1.49]	1.42 [0.77;2.62]	1.49 [0.81;2.72]	0.35	0.98	0.69
Log Insulin	214.4 [159.5;288.3]	187.5 [139.5;252.2]	270.5 [195.6;374.1]	238.3 [172.3;329.5]	0.33	0.19	0.97
IL-8	6.1 [4.9;7.4]	5.6 [4.4;6.9]	5.8 [4.4;7.2]	5.7 [4.4;7.1]	0.70	0.30	0.37
Log TNF-α	5.0 [4.0;6.2]	4.4 [3.5;5.5]	4.7 [3.4;6.1]	4.6 [3.6;5.8]	0.78	0.09	0.36
Klotho	4.9 [3.8;6.1]	4.3 [3.2;5.4]	3.8 [2.5;5.0]	5.9 [4.6;7.2]	0.18	0.18	0.02

All concentrations are in pg/ml, except for Klotho, which is ng/ml. The *p*-value for ‘Baseline’ shows differences between groups at baseline and ‘Training’ shows the differences after training as a whole group. ‘Training*intensity’ demonstrates an interaction between the training intensities in the cytokine and klotho concentration. Logarithmic transformation has been done to the variables indicated with ‘Log’. All data are presented as model based means [95% confidence interval, CI]. Logarithmic transformation has been done to the variables with ^. The values are LS means translated into original units.

Another well-established circulating cytokine is Leptin, which reflects adipose tissue mass^32^. Consistent with a significant decrease in adipose tissue mass (Table 1), both short-term exercise training programs decreased Leptin concentrations (Fig. 1b). We also determined the effects of short-term training on the concentrations of hepatocyte growth factor (HGF), a protein involved in angiogenesis, which was previously found to be increased after a single bout of HIIT exercise^33^. Short-term exercise training significantly reduced HGF concentrations to a similar extent in response to both training intensities (Fig. 1c). HGF is a key regulator of satellite cell activity during muscle regeneration^34^, and the reduction in HGF seen with short-term training may indicate training-related adaptations to skeletal muscle. Taken together, these data show that only two weeks of exercise training of both intensities decreases the cytokine concentrations of IL-6, HGF and Leptin. Interestingly, the reductions in these cytokines with training are in contrast with the increases that are seen in an acute exercise bout for IL-6^10,35^ and HGF^33^.

MCP-1 is a cytokine that exerts multiple biological functions, including the recruitment of monocytes^17^ and induction of angiogenesis^17,19^. Sprint interval training did not alter MCP-1 concentrations; however, moderate intensity training increased MCP-1 concentrations by 14% (Table 2, Fig. 1d). In contrast to changes in IL-6, HGF, Leptin, and MCP-1, moderate intensity training and sprint interval training did not significantly change NGF, insulin, IL-8, and TNF-α concentrations (Fig. 1e–h). Insulin concentrations decreased in the majority of subjects in both training intensities, but this was not statistically significant (Fig. 1f). We also determined the effects of exercise training intensity on Klotho concentrations, a soluble protein involved in anti-aging^26^. Moderate intensity training increased Klotho concentrations by 55%, while sprint interval training did not alter Klotho concentrations (Fig. 1i).

### MCP-1 and Klotho concentrations correlate with brown adipose tissue glucose uptake and metabolic parameters

**Fig. 2:**
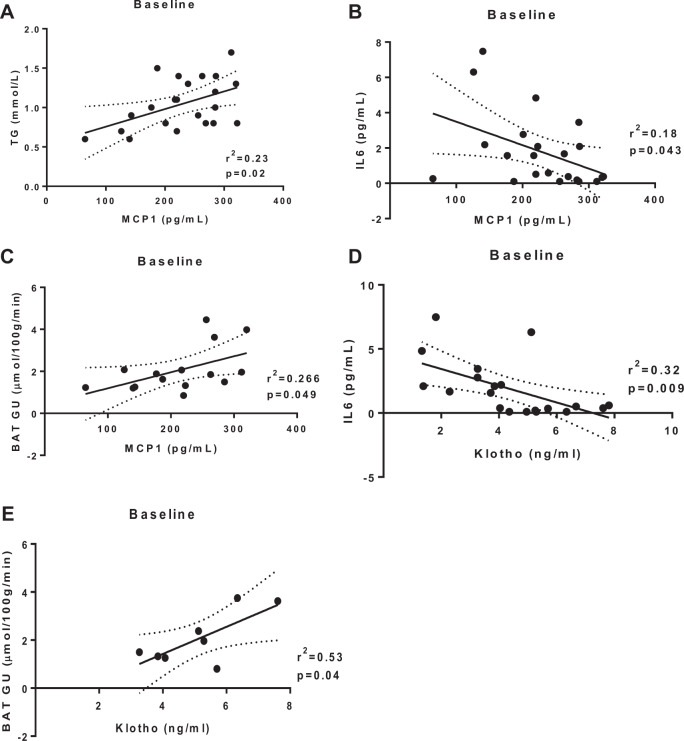
Correlations between baseline measurements of MCP-1 and Klotho and baseline metabolic parameters. Pearson’s correlations between baseline concentrations of MCP-1 (**a**–**c**) in both training groups with triglycerides (**a**), BAT glucose uptake (**b**) and IL-6 concentrations (**c**), and Klotho concentrations (**d**–**e**) with IL-6 concentrations (**d**) and BAT glucose uptake (**e**). *N* = 9–12/group.

We have previously shown that both short-term sprint interval training and moderate intensity exercise training increased insulin-stimulated glucose uptake into skeletal muscle and femoral white adipose tissue, whereas short-term training caused a decrease in glucose uptake into brown adipose tissue^1,26^. Given the intensity-dependent responses of MCP-1 and Klotho to short term training, we determined if there was a relationship between baseline concentrations of MCP-1 and Klotho and metabolic parameters using Pearson’s correlations. MCP-1 concentrations at baseline correlated positively with triglycerides, and MCP-1 showed a negative correlation with IL-6 concentrations (Fig. 2a, b). One of the advantages of this study is the availability of data on local glucose metabolism in different parts of the body, as determined by PET/CT-scanning^1^. At baseline, and prior to the start of the short-term training intervention, glucose uptake in brown adipose tissue correlated positively with MCP-1 concentrations (Fig. 2c). Similar to MCP-1, baseline measurements of Klotho correlated negatively with baseline IL-6 concentrations (Fig. 2d). There was no association between Klotho concentrations and skeletal muscle glucose uptake (data not shown). Klotho showed a strong positive correlation with brown adipose tissue glucose uptake (Fig. 2e).

### Exercise training-induced MCP-1 response correlates with changes in cytokines, HDL, and abdominal glucose uptake

**Fig. 3:**
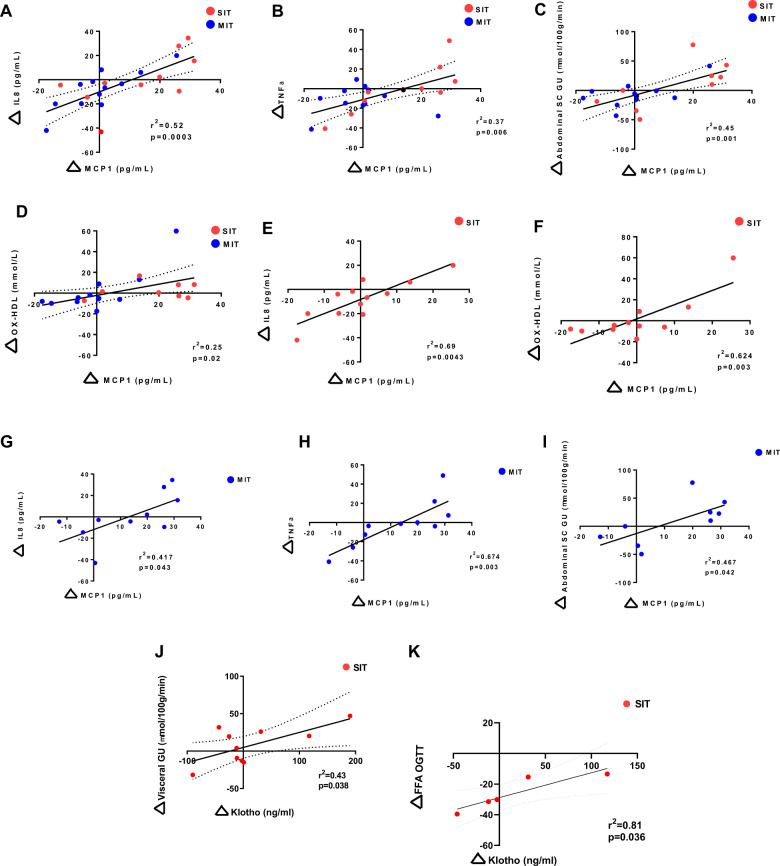
Correlations between change in MCP-1 and Klotho concentrations with change in metabolic parameters. Pearson’s correlations between change in concentrations of MCP-1 in both training groups with change in concentrations of IL8 (**a**), TNF-α (**b**), change in abdominal subcutaneous adipose tissue glucose uptake (**c**), and oxidized HDL (**d**) in both training groups combined. Correlation after sprint interval training (SIT) between change in MCP-1 concentrations and change in concentrations of IL8 (**e**) and Oxidized HDL (**f**). Correlation after moderate intensity training (MIT) between change in MCP-1 concentrations and change in concentrations of IL8 (**g**), TNF-α (**h**), and change in abdominal subcutaneous adipose tissue glucose uptake (**i**). Correlation after SIT training between change in Klotho concentrations and change in visceral subcutaneous adipose tissue glucose uptake (**j**) and free fatty acids measured during oral glucose tolerance test (**k**). *N* = 9–12/group.

Two weeks of short-term training reduced visceral fat mass and decreased the waist/hip ratio. To better understand the changes in MCP-1 concentrations after short-term exercise training and the relationship to responses in metabolic parameters, we next identified significant associations between the MCP-1 concentrations and all of the measured parameters, including anthropometrics, cytokine concentrations, glucose and lipid parameters, and in vivo glucose uptake before and after the training intervention. The response in MCP-1 concentrations after short-term exercise training correlated positively with the cytokines IL-8 and TNF-α (Fig. 3a, b). Changes in MCP-1 concentrations after training also correlated positively with abdominal subcutaneous glucose uptake, as determined by FDG-PET (Fig. 3c). In all subjects, changes in MCP-1 also showed a positive correlation with HDL (Fig. 3d).

While these significant correlations were seen when the two training programs were analyzed together, we next determined whether there were training intensity-specific correlations with MCP-1. For the sprint interval training, there was a positive correlation between MCP-1 and IL-8, and MCP-1 and HDL (Fig. 3e, f), but MCP-1 did not significantly correlate with TNF-α or subcutaneous abdominal glucose uptake (data not shown). With moderate intensity training, changes in MCP-1 correlated with IL-8, TNF-α, and subcutaneous abdominal glucose uptake (Fig. 3g–i). Taken together, these data demonstrate that responses in MCP-1 correlate with IL-8 and HDL in sprint interval training, and with IL-8, TNF-α, and abdominal subcutaneous glucose uptake after moderate intensity training. These data suggest that correlations between training response in MCP-1 and in other cytokines are dependent on exercise training intensity.

### Exercise training-induced changes in Klotho correlate with abdominal glucose uptake

Klotho concentrations after short-term exercise training in all subjects combined did not correlate significantly with any metabolic parameters, including change in weight and body fat mass or glucose uptake. However, when determining correlations by exercise intensity, in the sprint interval training group, Klotho correlated positively with visceral white adipose tissue glucose uptake (Fig. 3j). With moderate intensity training, there was no correlation between Klotho and visceral glucose uptake (data not shown). Sprint interval training also correlated with free fatty acid concentrations, as measured during the oral glucose tolerance test (Fig. 3k). Taken together, these data show that the effects of a short-term exercise training program on MCP-1 and Klotho concentrations, and the correlations between MCP-1 and Klotho with other cytokines and glucose metabolism measurements, are dependent on the intensity of the exercise training program.

## Discussion

Exercise training exerts a strong physiological stimulus on the body that results in the release of cytokines and other factors into the circulation, which in turn can regulate metabolic processes. Given the recent emphasis on short-term exercise training as a means to improve metabolism, in this study we determined if the intensity of short-term training was an important factor determining circulating factor responses in healthy men. While we found that both moderate and high intensity short-term training increased fitness and reduced the concentrations of IL-6, HGF, and Leptin, the circulating cytokine MCP-1 and Klotho responses were different between these different training intensities.

Our finding that short-term exercise training decreased IL-6 is consistent with animal data showing that exercise training reduced IL-6 concentrations in both normal glucose tolerant and hyperglycemic rats^36^. The decrease in IL-6 after short-term training is also in line with other training studies evaluating endurance training for longer periods of time including patients with coronary heart disease who trained for 12 weeks^37^ and healthy, older adults who trained for up to 10 months^38^. A short-term HIIT program in obese men did not significant reduce IL-6 concentrations, but did decrease IL-6 protein concentrations within subcutaneous white adipose tissue^39^, which is the primary tissue responsible for IL-6 production under resting conditions^15,40^. In contrast to exercise training studies generally showing decreases in IL-6, single bouts of endurance exercise have shown rapid increases in IL-6 immediately after exercise followed by a gradual decline in IL-6 concentrations back to baseline in the post-exercise period^10,35^. The IL-6 reduction seen after short-term training in our study thus appears to be more reflective of a longer-term training adaptation and may be indicative of a reduced pro-inflammatory state after exercise training.

The short-term moderate intensity exercise training program induced an intensity-specific increase in the concentrations of MCP-1, with only moderate intensity training showing an increase in this cytokine. MCP-1 can function to activate monocytes in skeletal muscle and play a role in muscle recovery after exercise^41^. As the duration of each of the six exercise bouts in the two week training programs was longer in the moderate intensity training program compared to the sprint interval training program (up to 60 vs. 27 min/day), the increased MCP-1 concentrations may reflect a prolonged muscle recovery after repeated exercise bouts in the moderate intensity training program. While we found no change in MCP-1 concentrations after sprint interval training, in a previous study, two weeks of HIIT in young, obese men reduced MCP-1 concentrations^39^, which may be due to their subjects being approximately 25 years younger (48 ± 5 years vs. 24 ± 5 years)^39^. MCP-1 concentrations increase with age^42^, and the baseline concentrations in our study were >50% higher.

We previously demonstrated that short-term training increases glucose uptake in femoral subcutaneous adipose tissue, which tended to be higher in sprint interval training compared to moderate intensity training ^1^. Here we show a positive correlation between the increase in abdominal glucose uptake and increase in MCP-1 concentrations after moderate intensity training. Exercise training causes a shift in adipose tissue macrophages from pro-inflammatory M1 to anti-inflammatory M2 macrophages^43^. It has been speculated that skeletal muscle-derived MCP-1 contributes to this shift to M2^44^. This raises the possibility that association between MCP-1 and increased abdominal adipose tissue glucose uptake may be due to changes in macrophages, although more studies are needed to better understand the role of MCP-1 in adipose tissue glucose uptake with exercise training.

Few studies have investigated Klotho in relation to acute exercise and exercise training^45^. A single exercise bout at maximal running capacity for 20 min increased Klotho concentrations in trained, healthy women, but not in men^28^. In another study, completing sixteen weeks of endurance training increased the response in Klotho concentrations after an acute exercise bout in young subjects (age 25–45), but the Klotho response to acute exercise was blunted in older subjects (age 65–74)^46^. A recent study found that 12-weeks of exercise training increases Klotho concentrations in middle-aged adults^47^. Our data now demonstrate that short-term moderate intensity exercise training for two weeks also regulates Klotho in men, and underscore that Klotho concentrations are responsive to a single bout of exercise, as well as short-term and long-term exercise training programs.

The increase in Klotho concentrations seen with sprint interval training correlated with increased visceral adipose tissue glucose uptake and baseline Klotho concentrations correlated with brown adipose tissue glucose uptake. The relationship between Klotho and visceral white adipose tissue and brown adipose tissue glucose metabolism is not known but could be mediated by fibroblast growth factors (FGFs). FGF-19 binds to Klotho, and overexpression of FGF-19 in mice activates brown adipose tissue thermogenesis^48^. These data also suggest that cytokine responses correlate to adipose tissue-depot specific responses in glucose uptake after exercise training, which highlights the heterogeneity of the different adipose tissue depots in the body.

The study also has limitations. Only men were studied, limiting the generalizability of the findings, and larger studies should include women, as well as men to determine if there are sex-specific differences. Subjects were metabolically healthy, and inclusion of subjects with obesity and type 2 diabetes can determine whether exercise-intensity targeted programs may mediate metabolic disease.

In summary, two weeks of exercise training at two different training intensities result in beneficial whole-body adaptations, which are accompanied by a decrease in IL-6, HGF and Leptin at both intensities. The intensity of short-term training is a key determinant in the regulation of the responses in MCP-1 and Klotho, as their concentrations increase with moderate intensity training only. These findings enhance our understanding of the effects of training intensity on cytokine responses and can contribute to the development of more personalized exercise training programs.

## References

[1] Motiani P (2017). Decreased insulin-stimulated brown adipose tissue glucose uptake after short-term exercise training in healthy middle-aged men. Diabetes Obes. Metab..

[2] Burgomaster KA (2008). Similar metabolic adaptations during exercise after low volume sprint interval and traditional endurance training in humans. J. Physiol..

[3] Vollaard NBJ (2009). Systematic analysis of adaptations in aerobic capacity and submaximal energy metabolism provides a unique insight into determinants of human aerobic performance. J. Appl. Physiol..

[4] Whyte LJ, Gill JMR, Cathcart AJ (2010). Effect of 2 weeks of sprint interval training on health-related outcomes in sedentary overweight/obese men. Metabolism.

[5] Richards JC (2010). Short-term sprint interval training increases insulin sensitivity in healthy adults but does not affect the thermogenic response to beta-adrenergic stimulation. J. Physiol..

[6] Milanović Z, Sporiš G, Weston M (2015). Effectiveness of high-intensity interval training (HIT) and continuous endurance training for VO2max improvements: a systematic review and meta-analysis of controlled trials. Sports Med..

[7] Stanford KI (2015). A novel role for subcutaneous adipose tissue in exercise-induced improvements in glucose homeostasis. Diabetes.

[8] Takahashi H (2019). TGF-β2 is an exercise-induced adipokine that regulates glucose and fatty acid metabolism. Nat. Metab..

[9] Leal LG, Lopes MA, Batista ML (2018). Physical exercise-induced myokines and muscle-adipose tissue crosstalk: a review of current knowledge and the implications for health and metabolic diseases. Front. Physiol..

[10] Pedersen BK, Febbraio MA (2008). Muscle as an endocrine organ: focus on muscle-derived interleukin-6. Physiol. Rev..

[11] Weisberg SP (2003). Obesity is associated with macrophage accumulation in adipose tissue. J. Clin. Invest..

[12] Nimmo MA, Leggate M, Viana JL, King JA (2013). The effect of physical activity on mediators of inflammation. Diabetes Obes. Metab..

[13] Febbraio MA, Pedersen BK (2005). Contraction-induced myokine production and release: is skeletal muscle an endocrine organ?. Exerc. Sport Sci. Rev..

[14] Oberbach A (2008). Long-term exercise training decreases interleukin-6 (IL-6) serum levels in subjects with impaired glucose tolerance: effect of the -174G/C variant in IL-6 gene. Eur. J. Endocrinol..

[15] Fischer CP (2006). Interleukin-6 in acute exercise and training: what is the biological relevance?. Exerc. Immunol. Rev..

[16] Deshmane SL, Kremlev S, Amini S, Sawaya BE (2009). Monocyte chemoattractant protein-1 (MCP-1): an overview. J. Interferon Cytokine Res..

[17] Wang T, Dai H, Wan N, Moore Y, Dai Z (2008). The role for monocyte chemoattractant protein-1 in the generation and function of memory CD8 T cells. J. Immunol..

[18] Takahashi M, Galligan C, Tessarollo L, Yoshimura T (2009). Monocyte chemoattractant protein-1 (MCP-1), not MCP-3, is the primary chemokine required for monocyte recruitment in mouse peritonitis induced with thioglycollate or zymosan A. J. Immunol..

[19] Salcedo R (2000). Human endothelial cells express CCR2 and respond to MCP-1: direct role of MCP-1 in angiogenesis and tumor progression. Blood.

[20] Low QE (2001). Wound healing in MIP-1alpha(-/-) and MCP-1(-/-) mice. Am. J. Pathol..

[21] Sell H, Dietze-Schroeder D, Kaiser U, Eckel J (2006). Monocyte chemotactic protein-1 is a potential player in the negative cross-talk between adipose tissue and skeletal muscle. Endocrinology.

[22] Zwetsloot KA, John CS, Lawrence MM, Battista RA, Shanely RA (2014). High-intensity interval training induces a modest systemic inflammatory response in active, young men. J. Inflamm. Res..

[23] Di Battista AP (2018). High-intensity interval training is associated with alterations in blood biomarkers related to brain injury. Front. Physiol..

[24] Barry JC, Simtchouk S, Durrer C, Jung ME, Little JP (2017). Short-term exercise training alters leukocyte chemokine receptors in obese adults. Med. Sci. Sports Exerc..

[25] Hu MC, Shiizaki K, Kuro-o M, Moe OW (2013). Fibroblast growth factor 23 and Klotho: physiology and pathophysiology of an endocrine network of mineral metabolism. Annu. Rev. Physiol..

[26] Kurosu H (2005). Suppression of aging in mice by the hormone Klotho. Science.

[27] Tan S-J (2018). High-intensity physical exercise increases serum α-klotho levels in healthy volunteers. J. Circulating Biomark..

[28] Santos-Dias A (2017). Longevity protein klotho is induced by a single bout of exercise. Br. J. Sports Med..

[29] Matsubara T (2014). Aerobic exercise training increases plasma Klotho levels and reduces arterial stiffness in postmenopausal women. Am. J. Physiol. Heart Circ. Physiol..

[30] Kiviniemi AM (2014). Cardiac autonomic function and high-intensity interval training in middle-age men. Med. Sci. Sports Exerc..

[31] Eskelinen J-J (2015). Muscle-specific glucose and free fatty acid uptake after sprint interval and moderate-intensity training in healthy middle-aged men. J. Appl Physiol..

[32] Shimizu H (1997). Serum leptin concentration is associated with total body fat mass, but not abdominal fat distribution. Int. J. Obes. Relat. Metab. Disord..

[33] Wahl P (2014). Effects of high intensity training and high volume training on endothelial microparticles and angiogenic growth factors. PLoS ONE.

[34] Chargé SBP, Rudnicki MA (2004). Cellular and molecular regulation of muscle regeneration. Physiol. Rev..

[35] Pedersen BK, Åkerström TCA, Nielsen AR, Fischer CP (2007). Role of myokines in exercise and metabolism. J. Appl. Physiol..

[36] Kim J-S (2014). Effect of exercise training of different intensities on anti-inflammatory reaction in streptozotocin-induced diabetic rats. Biol. Sport.

[37] Goldhammer E (2005). Exercise training modulates cytokines activity in coronary heart disease patients. Int. J. Cardiol..

[38] Kohut ML (2006). Aerobic exercise, but not flexibility/resistance exercise, reduces serum IL-18, CRP, and IL-6 independent of beta-blockers, BMI, and psychosocial factors in older adults. Brain Behav. Immun..

[39] Lira FS (2017). Short-term high- and moderate-intensity training modifies inflammatory and metabolic factors in response to acute exercise. Front. Physiol..

[40] Mohamed-Ali V (1997). Subcutaneous adipose tissue releases interleukin-6, but not tumor necrosis factor-alpha, in vivo. J. Clin. Endocrinol. Metab..

[41] Monteiro PA (2017). Modulation of inflammatory response arising from high-intensity intermittent and concurrent strength training in physically active males. Cytokine.

[42] Inadera H, Egashira K, Takemoto M, Ouchi Y, Matsushima K (1999). Increase in circulating levels of monocyte chemoattractant protein-1 with aging. J. Interferon Cytokine Res..

[43] Kawanishi N, Yano H, Yokogawa Y, Suzuki K (2010). Exercise training inhibits inflammation in adipose tissue via both suppression of macrophage infiltration and acceleration of phenotypic switching from M1 to M2 macrophages in high-fat-diet-induced obese mice. Exerc. Immunol. Rev..

[44] Catoire M, Kersten S (2015). The search for exercise factors in humans. FASEB J..

[45] Amaro-Gahete FJ (2018). Role of exercise on S-Klotho protein regulation: a systematic review. Curr. Aging Sci..

[46] Avin KG (2014). Skeletal muscle as a regulator of the longevity protein, Klotho. Front. Physiol..

[47] Amaro-Gahete FJ (2019). Exercise training increases the S-Klotho plasma levels in sedentary middle-aged adults: a randomised controlled trial. The FIT-AGEING study. J. Sports Sci..

[48] Kajimura S, Saito M (2014). A new era in brown adipose tissue biology: molecular control of brown fat development and energy homeostasis. Annu. Rev. Physiol..

